# Long-term cumulative associations of annual air pollution exposure and hospitalization with Parkinson’s Disease

**DOI:** 10.1097/EE9.0000000000000519

**Published:** 2026-08-10

**Authors:** Scott W. Delaney, Lauren Mock, Veronica A. Wang, Brad A. Racette, Gary W. Miller, Marianthi-Anna Kioumourtzoglou, Antonella Zanobetti, Danielle Braun, Daniel Mork

**Affiliations:** a Department of Environmental Health, Harvard T.H. Chan School of Public Health, Boston, Massachusetts; b Department of Biostatistics, Harvard T.H. Chan School of Public Health, Boston, Massachusetts; c Barrow Neurological Institute, Phoenix, Arizona; d Faculty of Health Sciences, University of the Witwatersrand, Johannesburg, South Africa; e Department of Environmental Health Sciences, Mailman School of Public Health, New York, New York; f Department of Data Science, Dana-Farber Cancer Institute, Boston, Massachusetts

**Keywords:** Air pollution, Parkinson’s disease, Medicare, Distributed lag model

## Abstract

**Background:**

Parkinson’s Disease (PD) prevention and treatment are complicated because biological processes underlying PD may begin several years before diagnosis. Previous studies suggest that increased exposure to fine particulate matter (PM_2.5_) and nitrogen dioxide (NO_2_) air pollution increases PD morbidity.

**Methods:**

We analyzed data from 10,366,083 Medicare fee-for-service beneficiaries (age 65+) in the contiguous United States from 2000 to 2016. We defined “hospitalization with PD” as a beneficiary’s first hospitalization claim with diagnosis codes indicating PD. We linked 10-year exposure histories for PM_2.5_, NO_2_, and summer ozone (O_3_). In a discrete-time survival analysis, we fitted distributed lag models and estimated the lagged associations between air pollution and the odds of first hospitalization with PD.

**Results:**

Increased PM_2.5_ and NO_2_ exposure at least 4 years before hospitalization was associated with increased odds of hospitalization with PD. Accounting for nonlinearities in exposure-response and 10 years of continuous exposure to PM_2.5_ at the 90th versus the 0.5th percentile (i.e., 11.8 μg/m^3^ vs. 3.0 μg/m^3^), the odds ratio for hospitalization with PD was 1.634 (95% CI: 1.489, 1.792). Similarly, for 10 years of continuous exposure to NO_2_ at the 90th versus the 0.5th percentile (i.e., 31.7 ppb vs. 3.7 ppb), the odds ratio for hospitalization with PD was 1.474 (95% CI: 1.379, 1.575). Evidence of a relationship between O_3_ exposure and odds for hospitalization with PD was more limited.

**Conclusions:**

Air pollution at least 4 years before hospitalization may increase the odds of hospitalization with PD. Reducing air pollution exposure may have long-term effects on PD prevention.

**What this study adds**

This work explores the cumulative risk of a decade of air pollution on hospitalization for Parkinson’s disease (PD). Previous work has primarily explored how a single time period of exposure is related to PD, missing risks from multiyear pollution exposure. We find that increased fine particulate matter and nitrogen dioxide exposure 4+ years prior raises the odds of Parkinson’s hospitalization with 10-year cumulative fine particulate matter and nitrogen dioxide exposures at 90th versus 0.5th percentile raising hospitalization odds by 63% and 47%, respectively. Nonlinear exposure-response suggests US Environmental Protection Agency air quality standards may not sufficiently protect against PD.

## Introduction

By 2030, Parkinson’s disease (PD) will afflict an estimated 1.3 million Americans, making it one of the fastest-growing neurodegenerative diseases.^1,2^ PD-related neurodegeneration leads to a wide range of disabling motor (e.g., bradykinesia, resting tremor, rigidity, and postural instability) and nonmotor symptoms (e.g., sleep problems, cardiac sympathetic denervation, and olfactory loss).^3–5^ Considering aging populations and limited disease-modifying therapies, clarifying environmental determinants of PD risk and progression is critical for reducing its public health and economic burden.

Genetic factors explain only up to one-third (i.e., 16%–36%) of PD risk,^6^ and growing evidence implicates environmental exposures in PD development.^7,8^ Ambient air pollutants, such as fine particulate matter (PM_2.5_), nitrogen dioxide (NO_2_), and ozone (O_3_), have been consistently linked to neurological disease outcomes.^8–16^ Mechanistic studies suggest that inhaling these pollutants may trigger systemic oxidative stress, neuroinflammation, disruption of the blood–brain barrier, and α-synuclein aggregation, processes implicated in PD pathogenesis.^17,18^ Characterizing the latency period by which air pollution exposure impacts PD is an essential step to further identify the interplay between environment and disease.

Epidemiological studies link long-term PM_2.5_ and NO_2_ exposure to increased PD incidence and progression, with less certainty for O_3_; however, meta-analyses highlight heterogeneity in this work due to differences in exposure assessment, populations, and study design, and the inherent limitations of observational data.^8,14–16^ Recent work using causal inference methods in a Medicare cohort similar to ours found long-term (annual) increases in PM_2.5_, NO_2_, and O_3_ were each associated with a higher risk of PD-related hospitalization.^10^ However, these studies largely rely on single time-point exposure (e.g., current or prior year) to investigate a disease that develops over decades. Longitudinal analyses with temporally resolved, multiyear exposure histories are needed to disentangle immediate, delayed, and cumulative effects and to assess whether regulatory standards adequately protect against PD.

The importance of understanding the impact of air pollution on health is underscored by the persistence of elevated PM2.5, NO2, and O3 levels in many regions, often above health-based standards.^19^ Mandated under the Clean Air Act, the US Environmental Protection Agency (EPA) establishes National Ambient Air Quality Standards (NAAQS), currently set at 9 µg/m^3^ (annual mean) for PM_2.5_, 53 ppb (annual mean) for NO_2_, and 70 ppb (8-hour mean) for O_3_.^20^ The World Health Organization (WHO) air quality guidelines (AQG) recommend even more stringent guidelines for safe exposure: 5 µg/m^3^ for PM_2.5_ (annual mean), 10 µg/m^3^ for NO_2_ (annual mean), and 60 ppb for O_3_ (peak season mean, 8-hour daily maximum).^21^ Evidence from mortality and cardiovascular disease studies, among other disease categories, indicates adverse effects even below current thresholds, raising concern that existing standards may not sufficiently protect against neurological outcomes such as PD.^22,23^

In this study, we assess immediate and delayed associations between long-term PM_2.5_, NO_2_, and O_3_ exposure and risk of PD-related hospitalization in a population-based cohort of 10,366,083 Medicare beneficiaries followed for 17 years. Using time-resolved exposure estimates, we evaluate cumulative and delayed effects and apply distributed lag models (DLMs) in a discrete-time survival framework. We interpret our findings relative to current EPA NAAQS and WHO AQG. Our objectives are to: (1) identify critical exposure windows preceding PD-related hospitalization; (2) determine whether PD risks emerge at pollutant concentrations below existing regulatory thresholds; and (3) inform air quality regulation and neuroprotective public health strategies.

## Methods

### Study population

We derived our sample from all adults 65 years of age or older living in the contiguous United States and enrolled in Medicare Part A fee-for-service (FFS) insurance from 2000 through 2016. Because all American citizens and many permanent residents are entitled to Medicare insurance at the age of 65 years, our study approximates a nationally representative sample for this population. For example, in 2000, 83% of all Medicare-eligible adults aged 65 and over were enrolled in FFS Medicare, and while FFS enrollment has declined somewhat over time, 66% of eligible adults were still enrolled in FFS Medicare by 2016.^24^ We obtained enrollment and hospitalization claims data from the Centers for Medicare and Medicaid Services, which included individual-level age, sex, race/ethnicity, Medicaid eligibility (a proxy for very low income), dates and diagnosis codes for each beneficiary’s hospitalizations, date of death (if applicable), and annually updated five-digit residential ZIP code.

Our analysis considers air pollution exposures up to a decade prior. We construct a longitudinal cohort of beneficiaries enrolled in Medicare FFS as of January 1, 2000, and consider all individuals who, as of December 31, 2009, (1) remained continuously enrolled in Medicare FFS insurance, (2) had no hospitalizations with PD, and (3) did not die. As a result, all beneficiaries in our cohort were at least 75 years of age at the beginning of 2010. We further restricted our sample to individuals with no missing exposure or covariate data (2.3% missingness). We define the time span encompassing 2000–2009 as the “lead-up period,” where we initiate measurements of past exposures, and 2010–2016 as the “study period.” Each beneficiary in our cohort was followed until they experienced a hospitalization with PD, died, or were censored by leaving Medicare FFS.

### Outcome definition

We defined “hospitalization with PD” as the first hospitalization claim including a PD-related International Classification of Diseases (ICD)-9 (332/332.0) or ICD-10 (G20) code within the first ten billing diagnosis code inputs. Due to limitations of inpatient claims data, we note our outcome is not a proxy for PD incidence and does not identify the initial diagnosis, which may have occurred in a prior outpatient setting. Rather, our outcome includes individuals who were hospitalized for a wide variety of reasons, including (but not limited to) fractures, psychosis, infections, and gastrointestinal complications, and also have an ICD-9/10 code for PD in any of the first ten diagnosis codes associated with a visit.^25^

### Exposure assessment

Between 2000 and 2016, we obtained daily exposure predictions for PM_2.5_, NO_2_, and O_3_ at a 1 km by 1 km grid resolution across the contiguous US based on previously validated models.^26–28^ For PM_2.5_ and NO_2_, we calculated annual exposures for each year of the study (i.e., 2000–2016) by averaging across daily predictions at each grid cell. For O_3_, we calculated annual exposures using 8-hour daily maximum values for summer days only (i.e., June through August). The exposure predictions were obtained from a well-validated ensemble learning approach and achieved a cross-validated R^2^ of 0.89, 0.84, and 0.86 for PM_2.5_, NO_2_, and O_3_, respectively; technical details of the exposure prediction models have been previously described and published.^26–28^

We calculated annual exposures for each ZIP code by averaging all annual exposures at grid centroids inside ZIP code boundaries to match the spatial resolution of the Medicare data. For each year of the study period, we assigned a rolling 10-year annual exposure history for every beneficiary matched at their historical ZIP code(s) of residence. For example, an individual in our study cohort in 2010 was assigned an exposure history for the years 2000–2009. If that same individual remained in the study cohort in 2016, they were assigned an exposure history for 2006–2015.

### Covariates

Age, Medicare-defined race/ethnicity, sex, Medicaid eligibility, and ZIP code of residence for each beneficiary were obtained from the Medicare Beneficiary Summary File, updated annually. We linked annually derived neighborhood-level demographic and socioeconomic variables derived from the 2000 US Census and 2011–2016 annual American Community Surveys. Demographic variables included ZIP code-level population density, percent of individuals who identify as Black or Hispanic, and percent of adults who graduated high school. Socioeconomic ZIP code-level variables included the median home price to income ratio, percent owner-occupied housing, and percent living below the federal poverty level. Linear interpolation was used to impute missingness in these variables.

### Statistical analyses

We adapted methods previously described in Mork et al.^29^ Specifically, to estimate the association between air pollution and first hospitalization with PD, we applied a discrete-time survival approach using multivariate logistic regression to accommodate time-varying exposure histories and confounding variables.^30^ We fit constrained DLMs that assumed a linear exposure–response relationship between the previous decade of air pollution exposure and hospitalization with PD. We also relaxed the linearity assumption in the DLM, using a distributed lag nonlinear model (DLNM) to consider potential nonlinear exposure–response relationships between exposure and outcome. For models assuming a nonlinear exposure–response relationship, we specified natural splines in the exposure dimension and placed knots at evenly spaced percentiles of pollutant exposure levels. For both DLM and DLNM (i.e., linear and nonlinear exposure–response relationships), we specified natural splines for the lag dimension with knots placed on the log scale across lagged time periods. Placing knots based on the log scale in the lag dimension enabled greater flexibility at more proximate lags and reflected the assumption that more recent pollutant exposure may have greater impacts on hospitalization with PD than exposure further in the past. Following Mork et al^29^, we defined spline bases allowing for 3 degrees of freedom in the lag dimension and 5 degrees of freedom in the exposure concentration dimension (i.e., knots at boundaries and 25th, 50th, and 75th percentiles), which regularizes the model to accommodate the temporal autocorrelation in year-to-year exposure variation and, for the DLNM, provides ample flexibility for a nonlinear exposure–response relationship.

To account for the competing risk of censoring via death, we used stabilized inverse probability weights. We assumed censoring (e.g., by leaving Medicare FFS for a Medicare Advantage plan) was independent of air pollution exposure. We adjusted all models for both individual-level (age, sex, race/ethnicity, and Medicaid eligibility) and area-level (population density, percent Black or Hispanic, percent graduated high school, percent owner-occupied housing, home price to income ratio, and percent living below the poverty level) covariates to address possible confounding. As increased age is highly correlated with worsening health conditions, we specified age as a quadratic term to account for a potentially nonlinear association. All models also included a fixed effect for beneficiary state of residence to account for potential unmeasured spatial confounding (e.g., state medical billing incentives and climate types) that was not captured through other area-level covariates, and a fixed effect for each year of follow-up to further account for longer-term temporal trends.

To interpret results from models that assumed linear exposure–response functions, we calculated odds ratios (ORs) comparing exposure levels required by EPA NAAQS to those recommended by the WHO, which promulgates more conservative AQGs for similar public health purposes. A change from AQG to NAAQS amounted to increases of 4 μg/m^3^ PM_2.5_, 43 ppb NO_2_, and 10 ppb summer O_3_. To interpret results from models that assumed nonlinear exposure–response functions, we calculated ORs comparing exposure at prespecified values (e.g., certain percentiles of the exposure distributions, AQG recommendations, and NAAQS requirements) to exposure at the 0.5th percentile of the exposure distribution for each pollutant.

#### Sensitivity analyses

To assess the robustness of our results, we conducted two sensitivity analyses. First, using the same study design, we fit DLMs assuming a linear exposure–response function using only 5 years of exposure history (vs. 10 years in the primary analyses) to assess whether lagged effect estimates were robust to the length of exposure history considered. For the lag-response relationship considering 5 years of exposure history, we fit splines with 2 degrees of freedom to achieve similar flexibility in the lag dimension compared with our primary model. Second, to explore potential confounding bias in our primary models, we re-ran primary models with several additional covariates, including county-specific smoking rates and mean body mass index, and ZIP code-specific summer and winter mean maximum temperatures and minimum relative humidity.

#### Stratified analysis

Since previous studies suggest that the adverse health effects of PM_2.5_ may vary across urban and rural areas,^31,32^ we refit the primary models stratified by urbanicity. We determined urbanicity using the US Department of Agriculture’s Rural-Urban Commuting Area Codes, classifying those living in ZIP codes assigned to codes 1–3 (metropolitan) as urban and those living in ZIP codes assigned to the remaining codes 4–10 (nonmetropolitan) as rural.^33^

The Human Subjects Committee at the Harvard T. H. Chan School of Public Health approved this study. It was exempt from informed consent requirements due to the use of existing administrative data. All methods were performed in accordance with the relevant guidelines and regulations. We conducted all analyses using R software version 4.2.2 (R Foundation for Statistical Computing, Vienna, Austria). Code to reproduce all analyses is publicly available at https://github.com/NSAPH-Projects/air-pollution_pd_distributed-lag.

## Results

### Sample characteristics and exposure distributions

Our sample included 10,366,083 unique beneficiaries contributing 156,484,165 person-years of data. (See Supplementary Figure 1; https://links.lww.com/EE/A456 for an inclusion/exclusion diagram.) During the study period, 172,238 (1.7%) beneficiaries experienced a first hospitalization with PD; among these beneficiaries’ first hospitalizations with PD, 4.1% had a PD-related ICD code listed as the first code in the claim and 12.2% had a PD-related ICD code listed as the second code in the claim. The remaining hospitalization outcomes had PD-related ICD codes among the third through tenth codes in the claim. The mean age at first hospitalization with PD was 84.1 years. Beneficiaries who experienced a hospitalization with PD (vs. the full study population) were more likely to be White (91.4% vs. 89.3%) and male (54.0% vs. 38.6%) (Table 1). There was a weak to moderate correlation between 10-year average exposures: Cor(PM_2.5_, NO_2_) = 0.40; Cor(PM_2.5_, O_3_) = 0.33; Cor(NO_2_, O_3_) = 0.28.

**Table 1.** Characteristics of Medicare beneficiaries included in the study sample, 2000–2018.

Characteristic	All Beneficiaries	Hospitalization With PD^b^
Total	10,193,845	172,238
Sex, % of col.^a^		
Male	38.6	54.0
Female	61.4	46.0
Age in years, % of col.		
75–79	37.2	37.9
80–84	30.8	36.0
85–89	20.3	20.1
90–94	8.3	5.2
95+	3.4	0.8
Racial or ethnic identity, % of col.		
White	89.3	91.4
Black	6.5	4.7
Asian	1.5	1.4
Hispanic	1.5	1.6
Native American	0.3	0.3
Other	0.8	0.7
Medicaid eligibility, % of col.		
Ineligible	87.2	87.6
Eligible	12.8	12.4

a Percentages represent the percentage of beneficiaries with a given characteristic within each column.

b Beneficiaries classified as “with hospitalization with PD” are those who experienced a first hospitalization during the study period (2010–2016) that resulted in a Medicare claim including a PD diagnosis code. All other beneficiaries are classified as “without hospitalization with PD.”

During the lead-up and study period (i.e., from 2000 to 2016), the median average annual PM_2.5_ exposure was 9.6 μg/m^3^, which was higher than the current NAAQS for annual PM_2.5_ (9 μg/m^3^) and the current AQG (5 μg/m^3^) (Table 2). The median exposure for average annual NO_2_ was 14.8 ppb, which was well below the current NAAQS of 53 ppb but above the current AQG of 10 ppb. For summer O_3_, the median annual exposure was 42.5 ppb, which was below both the current NAAQS (70 ppb) and AQG (60 ppb).

**Table 2.** Distribution of annual average exposure concentrations for the three pollutants in this study.

	Percentiles of Pollutant Distribution								
Pollutant	0.5%	10%	25%	50%	75%	90%	99.5%	AQG^a^	NAAQS^b^
PM_2.5_ (μg/m^3^)	3.0	6.5	8.0	9.6	10.7	11.8	16.2	5.0	9.0
NO_2_ (ppb)	3.7	7.0	9.9	14.8	22.6	31.7	43.4	10.0	53.0
O_3_ (ppb)^c^	24.5	35.1	38.9	42.5	45.7	59.6	65.3	60.0	70.0

a AQG values are the World Health Organization Air Quality Guideline targets for average annual exposure.

b NAAQS values are US Environmental Protection Agency long-term (annual) primary National Ambient Air Quality Standards for each pollutant.

c Average 8-hour maximum summer ozone.

### PM_2.5_

**Figure 1.**
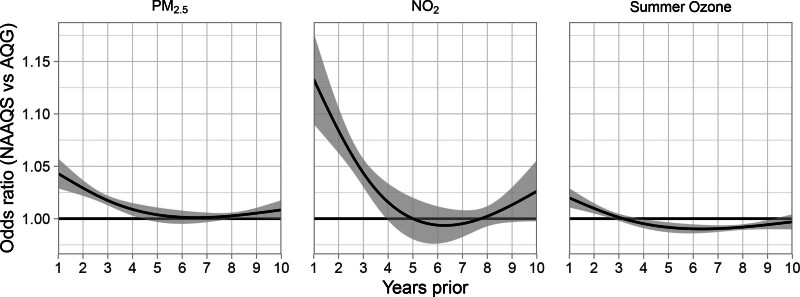
Lag-response linear relationship comparing annual air pollutant exposure to NAAQS versus AQG levels. Plotted are distributed lag odds ratios (*y* axis) with 95% confidence intervals (shaded area) from 1 to 10 prior years (*x* axis) due to a change from AQG to NAAQS levels (Table 2) in each pollutant. See Supplementary Table 1; https://links.lww.com/EE/A456 for a numerical table of estimates.

The linear DLM indicates that increased PM_2.5_ air pollution exposure from up to 4 years prior was associated with an increased risk of hospitalization with PD. Based on the linear DLM, the OR of hospitalization with PD in the first year (lag 1) following exposure to an annual average PM_2.5_ concentration of 9 μg/m^3^ (the current long-term NAAQS) versus 5 μg/m^3^ (the current AQG) was 1.043 (95% CI: 1.029, 1.057) (Figure 1, Supplementary Table 1; https://links.lww.com/EE/A456). Notably, the NAAQS for PM_2.5_ (9 μg/m^3^) corresponds to the 39th percentile of the PM_2.5_ distribution, while the AQG (5 μg/m^3^) corresponds to the 4th percentile. Put differently, this contrast represents the OR for hospitalization risk comparing annual exposure at the 39th versus the 4th percentile of the PM_2.5_ exposure distribution.

**Figure 2.**
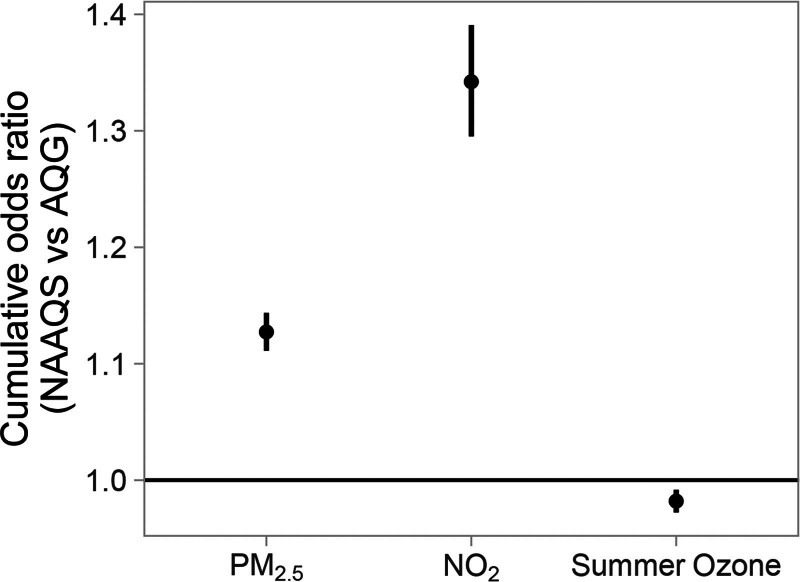
Ten-year cumulative association between air pollutant exposure and risk of hospitalization with PD. Plotted are cumulative odds ratios for each pollutant with 95% confidence intervals, comparing odds at NAAQS versus AQG (Table 2) for each pollutant. Cumulative ORs are calculated by summing the distributed lag estimates over the entire exposure time span.

The lag-response curve in Figure 1 suggests that the effects of elevated PM_2.5_ pollution exposure persist for at least 4 years. Both OR point estimates and the lower bounds of confidence intervals were above the null value of 1.0 through lag year 4 (Figure 1, Supplementary Table 1; https://links.lww.com/EE/A456). In lag years 5–10, the results suggested no association between PM_2.5_ exposure and the risk of hospitalization with PD. Thus, the full 10-year lag-response curve suggested that associations between increased PM_2.5_ exposure and increased risk of hospitalization with PD were present at only proximate (i.e., lag years 1–4) time periods after initial exposure. Based on the linear DLM, the cumulative OR of hospitalization with PD after 10 years of continuous exposure to annual average PM_2.5_ concentrations at NAAQS (9 μg/m^3^) versus AQG (5 μg/m^3^) was 1.127 (95% CI: 1.111, 1.144) (Figure 2, Supplementary Table 1; https://links.lww.com/EE/A456).

**Figure 3.**
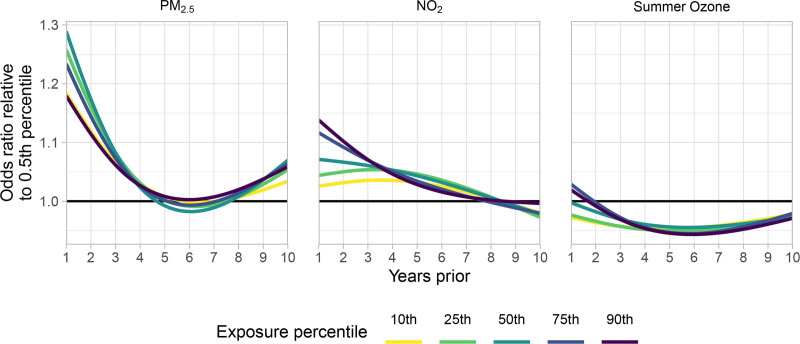
Lag-response relationship at multiple air pollutant exposure levels. Plotted are distributed lag nonlinear effect estimates at five different percentiles (exposure-concentration percentiles given in Table 2) compared with exposure at the 0.5th percentile of each pollutant’s exposure distribution. The odds ratio (*y* axis) at each percentile is shown at 1–10 prior years of exposure (*x* axis) for each pollutant.

Figure 3 plots lag-response curves at various percentiles of the PM_2.5_ exposure distribution (relative to the 0.5th percentile, 3.0 μg/m^3^) as estimated by the DLM with a nonlinear exposure–response function. Overall, we observed similar lag-response patterns between the linear and nonlinear models, both indicating that higher exposure in the proximal lags has a larger impact on the odds of hospitalization with PD. Distributed lag ORs at the 10th percentile of PM_2.5_ exposure (6.5 μg/m^3^) were generally lower compared with lagged ORs at higher levels of exposure, though point estimates for ORs at more proximal lags were similar for the 10th and 90th percentiles.

**Figure 4.**
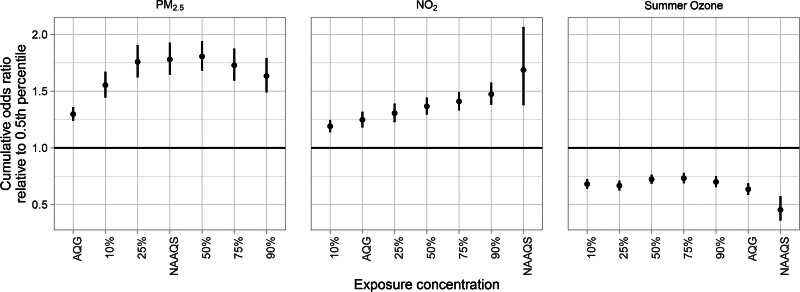
Distributed lag nonlinear model, 10-year cumulative associations between air pollutant exposure and risk of hospitalization with PD at multiple concentrations. Plotted are cumulative odds ratios and 95% confidence intervals at five different exposure concentration percentiles, AQG, and NAAQS (Table 2) for each of three pollutants from the distributed lag model assuming nonlinear exposure–response relationships. Cumulative ORs are calculated by summing across time the distributed lag nonlinear effects taken at the corresponding exposure concentration compared with the 0.5th percentile of each pollutant’s exposure distribution. See Supplementary Table 2; https://links.lww.com/EE/A456 for numerical estimates.

Figure 4 plots cumulative ORs after 10 years of continuous PM_2.5_ air pollution exposure at various percentiles of the PM_2.5_ distribution (versus the 0.5th percentile) from the nonlinear DLM. ORs for the risk of hospitalization with PD increased from low concentrations of PM_2.5_ up until the 25th percentile of the PM_2.5_ distribution (i.e., 8.0 μg/m^3^), and long-term exposure to concentrations above that level did not appear to confer additional risk. Thus, compared with a person exposed to 3.0 μg/m^3^ (the 0.5th percentile), a person exposed to 5 μg/m^3^ (the AQG recommendation) over the past decade would have a cumulative OR of 1.297 (95% CI: 1.238, 1.360) for first hospitalization with PD, whereas someone exposed to the long-term NAAQS for PM_2.5_ of 9 μg/m^3^ over the past decade would have an OR of 1.779 (95% CI: 1.644, 1.927; Supplementary Table 2; https://links.lww.com/EE/A456). However, exposure to concentrations beyond 9 μg/m^3^ of PM_2.5_ does not continue to see significant increases in OR; for example, exposure at the 75th percentile (10.7 μg/m^3^) has an OR of 1.728 (95% CI: 1.593, 1.874).

### Nitrogen dioxide

Based on linear DLM, long-term exposure to increased NO_2_ pollution was also associated with an increased risk of hospitalization with PD (Figure 1). The estimated OR for prior year (lag 1) exposure to the NAAQS for NO_2_ (53 ppb, corresponding to the 99.9th percentile of the NO_2_ distribution) versus the AQG for NO_2_ (10 ppb, corresponding to the 26th percentile) was 1.133 (95% CI: 1.090, 1.177) (Supplementary Table 1; https://links.lww.com/EE/A456). The lag-response curve suggests the increased risk associated with increased NO_2_ exposure persists through lag year 3 before lagged ORs return to null values in lag years 4–10. However, lagged OR point estimates reveal an overall “J” shape of the lag-response curve, such that point estimates increase above 1.0 at more distal lags (e.g., lags 9–10), even while confidence intervals widen and include the null value. The cumulative, 10-year OR comparing annual average NO_2_ exposure at the NAAQS (53 ppb) versus the AQG (10 ppb) was 1.342 (95% CI: 1.295, 1.391) (Figure 2, Supplementary Table 1; https://links.lww.com/EE/A456).

Figure 3 compares lag-response curves for exposure at different percentiles of the NO_2_ distribution versus the 0.5th percentile (3.7 ppb) of the distribution based on the DLNM analysis and shows a linear trend across lags toward the null value. At more proximal lag years (e.g., lags 1–3), ORs are higher when exposure is greater, after which ORs at various exposure percentiles converge and increase together toward the null value (Figure 3). The cumulative OR of hospitalization with PD from DLNM, given exposure at the 90th versus 0.5th percentile (31.7 vs. 3.7 ppb) for NO_2_ is 1.474 (95% CI: 1.379, 1.575), while the corresponding estimate given exposure at the 10th versus 0.5th percentile (7.0 vs 3.7 ppb) NO_2_ is 1.191 (95% CI: 1.138, 1.246) (Figure 4, Supplementary Table 2; https://links.lww.com/EE/A456).

### Ozone

In contrast to findings for PM_2.5_ and NO_2_ exposure, evidence suggesting an association between increased O_3_ exposure and increased risk of hospitalization with PD was more limited. From the linear DLM, comparing annual exposure to the NAAQS for O_3_ (70 ppb) versus the AQG (60 ppb), the lag-response curve in Figure 1 suggests the risk of hospitalization with PD is elevated in lag years 1 and 2. For example, the estimated OR for hospitalization with PD, going from AQG to NAAQS O_3_ exposure, was 1.020 (95% CI 1.011, 1.029) in lag year 1 and 1.010 (95% CI 1.005, 1.015) in lag year 2 (Supplementary Table 1; https://links.lww.com/EE/A456). However, ORs in lag years 3 and 4 suggest no association, while ORs from lag years 5 through 9 suggest increased O_3_ exposure is associated with decreased odds of hospitalization with PD. Based on linear DLM, the cumulative OR for hospitalization with PD after 10 years of exposure to the NAAQS versus the AQG for O_3_ was 0.982 (95% CI 0.972, 0.992) (Figure 2, Supplementary Table 1; https://links.lww.com/EE/A456). Lag-response curves based on DLNM analysis (Figure 3) suggest the highest exposures (e.g., 75th and 90th) were associated with increased risk of hospitalization with PD in lag year 1 only. All other estimates in Figure 3 suggest either null or inverse associations. DLNM cumulative 10-year ORs (Figure 4) suggested an inverse relationship between long-term O_3_ exposure and risk of hospitalization with PD.

### Sensitivity analyses

Results from sensitivity analyses (5-year exposure history and additional area-level ecological covariates) were consistent with findings from primary models (Supplementary Figure 2; https://links.lww.com/EE/A456).

### Stratified analysis

For models stratified by urbanicity, lag-response curves differed notably for urban and rural regions, although confidence intervals were wide (Supplementary Figure 3; https://links.lww.com/EE/A456). Cumulative effects comparing the NAAQS and the AQG suggested no association between hospitalization with PD and any of the three air pollutants in rural or urban regions (Supplementary Figure 4; https://links.lww.com/EE/A456).

## Discussion

To the best of our knowledge, our study is the first to estimate the 10-year lagged association between air pollution exposure and hospitalization with PD. In doing so, it provides insight into the time horizon over which biological processes linking air pollution to PD morbidity may operate. Our study finds that increases from the 0.5th to 90th percentiles of exposure to PM_2.5_ and NO_2_ are associated with 1.634 and 1.474 higher odds of hospitalization with PD, respectively. Our study has three main takeaways. First, we find that the effects of PM_2.5_ and NO_2_ may last approximately 4 years after the year of initial exposure, a time horizon considerably longer than is captured in prior studies assessing same- or prior-year exposure only. Second, we find no evidence suggesting that increased long-term O_3_ exposure exacerbates the risk of hospitalization with PD. Third, our results suggest that the current primary annual NAAQS for both PM_2.5_ and NO_2_ may not be sufficiently low to protect the public’s health with an adequate margin of safety, as is required by law.

Lag-response curves reported in this study for PM_2.5_ and NO_2_ provide evidence that biological processes underlying previously reported relationships between air pollution exposure and PD, which are also supported by mechanistic and toxicological studies,^34,35^ may begin several years before diagnosis. Specifically, increased pollutant exposure is associated with an increased risk of hospitalization with PD, and the risk decreases over time until approximately year 5 with some indication of longer effects due to extended PM_2.5_ exposure. The upward slope of the PM_2.5_ lag-response curve at distal lags may also be consistent with “harvesting,” wherein hospitalizations for high-risk beneficiaries are advanced in time due to PM_2.5_ exposure, leading to a temporary depletion of the risk pool. This phenomenon most frequently occurs in studies of short-term, acute exposures but was also observed in long-term relationships between PM_2.5_ air pollution and Alzheimer disease and related dementia.^29^ Although lag effects indicate the previous 4 years of exposure are more impactful, evidence of impacts from earlier exposure periods may be limited due to statistical power; in this case, reporting a total cumulative effect from the entire time period is preferable.^36^ Our findings are directly relevant to future EPA regulatory decision-making. Specifically, the EPA periodically conducts policy and risk assessments to determine whether current NAAQS limits are adequate to protect public health. In prior policy assessments, the EPA has concluded that evidence of associations between PM_2.5_ and NO_2_ and PD morbidity was insufficient to incorporate into its analyses. Our study adds to the existing literature and bolsters the evidence base suggesting that decreased air pollution exposure may reduce PD morbidity among older adults in the United States. Importantly, our findings suggest the current annual NAAQS for PM_2.5_ may not be low enough to protect adults from the impact of PM_2.5_ on PD disease processes.

The inverse relationship reported in this study between long-term O_3_ exposure and the risk of hospitalization with PD is not consistent with known biological mechanisms. A recent review highlighted the biological mechanisms through which O_3_ pollution induces chronic oxidative stress and loss of inflammatory response regulation in both the brain and the intestine, contributing to the neurodegeneration seen in PD.^37^ However, multiple prior epidemiological studies have reported allegedly protective relationships between long-term O_3_ exposure and multiple health outcomes, including those related to neurodegenerative diseases.^8^ This is despite robust evidence showing that increased long-term O_3_ exposure is associated with increased premature morbidity.^38^ Thus, future studies should investigate whether previously reported inverse relationships for O_3_ are due to confounding (e.g., climate type) or some other form of bias, possibly due to the complex relationships between O_3_ and other air pollutants. For example, future work might consider lagged multiple pollutant models that account for relationships across components.^39^

In addition to our three main findings, these results also contribute more broadly to the literature exploring the effects of long-term air pollution on various measures of PD morbidity and mortality. Our results are consistent with a recent meta-analysis from Xie et al^8^, who reported consistent harmful associations between long-term PM_2.5_ exposure (17 studies) and long-term NO_2_ exposure (11 studies) and PD-related outcomes. The same meta-analysis found no association between long-term O_3_ and PD outcomes based on three studies. Wang et al^10^, whose results were not included in the Xie et al^8^ meta-analysis, reported harmful associations between each of PM_2.5_, NO_2_, and O_3_ and the risk of hospitalization with PD. The Wang et al^10^ study considered only a single year of exposure and linear exposure–response relationships, and in fact, their O_3_ results are similar to the lag 1 linear model results found in our study. Finally, our investigation of the role of urbanicity in the relationship between PM_2.5_ exposure and hospitalization with PD produced substantially different results in urban vs. rural areas, suggesting that more research is needed to understand which PM_2.5_ components are most strongly associated with PD morbidity.

Our study has several limitations. First, our study design required a 10-year lead-up period, and as a result, it is generalizable only among beneficiaries older than 75 years. The outcome definition also excludes individuals with PD who were never hospitalized; however, we have high confidence that the hospitalization outcome is the first on record due to the 10-year clean lead-up period. Second, the exposure assessment may not be representative of individual exposure and has the potential for measurement error. However, the exposure models showed excellent predictive accuracy and are not expected to depend on the hospitalization outcome, as the models predicting exposure were fit independently. The presence of non-differential measurement error could result in bias towards the null.^40^ Third, our specific outcome, first hospitalizations with PD diagnosis codes, should not be interpreted as a PD diagnosis. Within this definition, individuals with a PD diagnosis who were not hospitalized were considered to not have experienced the outcome, and this may disproportionately include minorities or individuals without access to care. Outcome misclassification could result from clinical misdiagnosis or hospital billing errors. The hospitalization outcome may also reflect disease severity and healthcare access.^41^ Fourth, Medicare records do not include individual-level behavior data such as smoking and alcohol consumption, obesity, genetic factors, or occupational data that may modify, mediate, or confound the relationships of interest, and family history of PD was unavailable. Additionally, limitations in the methodology could include a misspecified outcome model and oversmoothing of the lag-response relationship. However, given the long-term development of the disease, we expect increased smoothness in the lag-response patterns for exposure. Our model adjusted for a broad spectrum of individual and area variables that have been shown in previous air pollution studies to sufficiently account for possible confounding.^42^ Our study also controlled for ambient seasonal maximum temperatures, which have been shown to directly relate to increased PM_2.5_ levels and acute risk of hospitalization with PD.^43,44^ Finally, we assumed no relationship between air pollution and beneficiaries leaving Medicare FFS; regardless, we expect any dependencies in the censoring mechanism to have a negligible impact on the results.

Long-term exposure to PM_2.5_ and NO_2_ air pollution may pose a threat to adults living with PD, and this threat may persist for 4 years after exposure. Current regulations and guidelines (US EPA NAAQS and WHO AQG) may be insufficient to protect older adults living with PD from the harms of increased air pollution, and more stringent regulations may lead to less PD-related morbidity. Clinicians and policymakers should consider the long-term impact of air pollution among patients with PD and devise strategies to mitigate these risks.

## Conflicts of interest statement

The authors declare that they have no conflicts of interest with regard to the content of this report.

## ACKNOWLEDGMENTS

The computations in this paper were run on the FASSE cluster supported by the FAS Division of Science Research Computing Group at Harvard University and the Research Computing Environment supported by the Institute for Quantitative Social Science in the Faculty of Arts and Sciences at Harvard University.

## Supplementary Material


