# Supplementary material for: Long-term cumulative associations of annual air pollution exposure and hospitalization with Parkinson’s Disease
Source: Environ Epidemiol. 2026 Aug 10;10(4):e519. doi: 10.1097/EE9.0000000000000519 (PMC13461014; doi:10.1097/EE9.0000000000000519)
Supplement: Supplementary file 1 [file ee9-10-e519-s001.pdf]

Long-term cumulative associations of annual air pollution exposure and hospitalization with Parkinson's Disease

Scott W. Delaney, ScD JD MPH; Lauren Mock, MS; Veronica Wang, PhD; Brad A. Racette, MD; Gary W. Miller, PhD; Marianthi-Anna Kioumourtzoglou, ScD MSPH; Antonella Zanobetti, PhD; Danielle Braun, PhD; Daniel Mork, PhD

Supplementary Materials

Closely related papers.

Supplement Table 1. Distributed lag linear model OR estimates and 95% confidence intervals comparing NAAQS to WHO AQG.
Supplement Table 2. Cumulated distributed lag nonlinear model OR estimates and 95% confidence intervals for selected quantile contrasts compared to the 0.5th percentile of exposure distribution

Supplement Figure 1. Study population inclusion/exclusion diagram.

Supplement Figure 2. Distributed lag linear model odds ratios for sensitivity analyses.

Supplement Figure 3. Lag-response linear relationship comparing annual air pollutant exposure to NAAQS versus AQG levels, stratified by urbanicity.

Supplement Figure 4. 10-year cumulative association between air pollutant exposure and risk of hospitalization with PD, stratified by urbanicity.

Closely related papers to this work include:

- Mork, D., Braun, D. and Zanobetti, A., 2023. Time-lagged relationships between a decade of air pollution exposure and first hospitalization with Alzheimer's disease and related dementias. Environment international, 171, p.107694.
- Wang, V.A., Delaney, S., Flynn, L.E., et. al., 2024. The effect of air pollution on hospitalizations with Parkinson’s disease among medicare beneficiaries nationwide. npj Parkinson's Disease, 10(1), p.196.

Supplement Table 1. Distributed lag linear model OR estimates and 95% confidence intervals comparing NAAQS to WHO AQG.

Years prior	$PM_{2.5}$	NO_2	Summer O_3
1	1.043 (1.029, 1.057)	1.133 (1.090, 1.177)	1.020 (1.011, 1.029)
2	1.029 (1.022, 1.037)	1.084 (1.063, 1.106)	1.010 (1.005, 1.015)
3	1.017 (1.013, 1.022)	1.044 (1.030, 1.057)	1.001 (0.998, 1.005)
4	1.009 (1.003, 1.015)	1.016 (0.998, 1.033)	0.995 (0.991, 1.000)
5	1.004 (0.997, 1.011)	1.000 (0.980, 1.020)	0.992 (0.987, 0.997)
6	1.001 (0.995, 1.008)	0.994 (0.976, 1.012)	0.990 (0.986, 0.995)
7	1.001 (0.997, 1.006)	0.995 (0.983, 1.008)	0.991 (0.987, 0.994)
8	1.003 (1.000, 1.006)	1.002 (0.993, 1.012)	0.992 (0.989, 0.995)
9	1.005 (1.000, 1.010)	1.013 (0.997, 1.030)	0.994 (0.990, 0.999)
10	1.008 (0.999, 1.018)	1.026 (0.998, 1.056)	0.997 (0.990, 1.004)
Cumulative	1.127 (1.111, 1.144)	1.342 (1.295, 1.391)	0.982 (0.972, 0.992)

Supplement Table 2. Ten-year cumulative distributed lag nonlinear model OR estimates and 95% confidence intervals for selected quantile contrasts compared to the 0.5th percentile of exposure distribution.

Quantile	$PM_{2.5}$	NO_2	Summer O_3
10%	1.553 (1.443, 1.671)	1.191 (1.138, 1.246)	0.682 (0.640, 0.726)
25%	1.758 (1.621, 1.906)	1.306 (1.227, 1.390)	0.667 (0.625, 0.711)
50%	1.806 (1.680, 1.941)	1.367 (1.294, 1.443)	0.722 (0.684, 0.764)
75%	1.728 (1.593, 1.874)	1.410 (1.331, 1.493)	0.733 (0.689, 0.780)
90%	1.634 (1.489, 1.792)	1.474 (1.379, 1.575)	0.700 (0.654, 0.750)
NAAQS	1.779 (1.644, 1.927)	1.686 (1.376, 2.066)	0.454 (0.359, 0.575)
AQG	1.297 (1.238, 1.360)	1.248 (1.180, 1.319)	0.636 (0.586, 0.690)

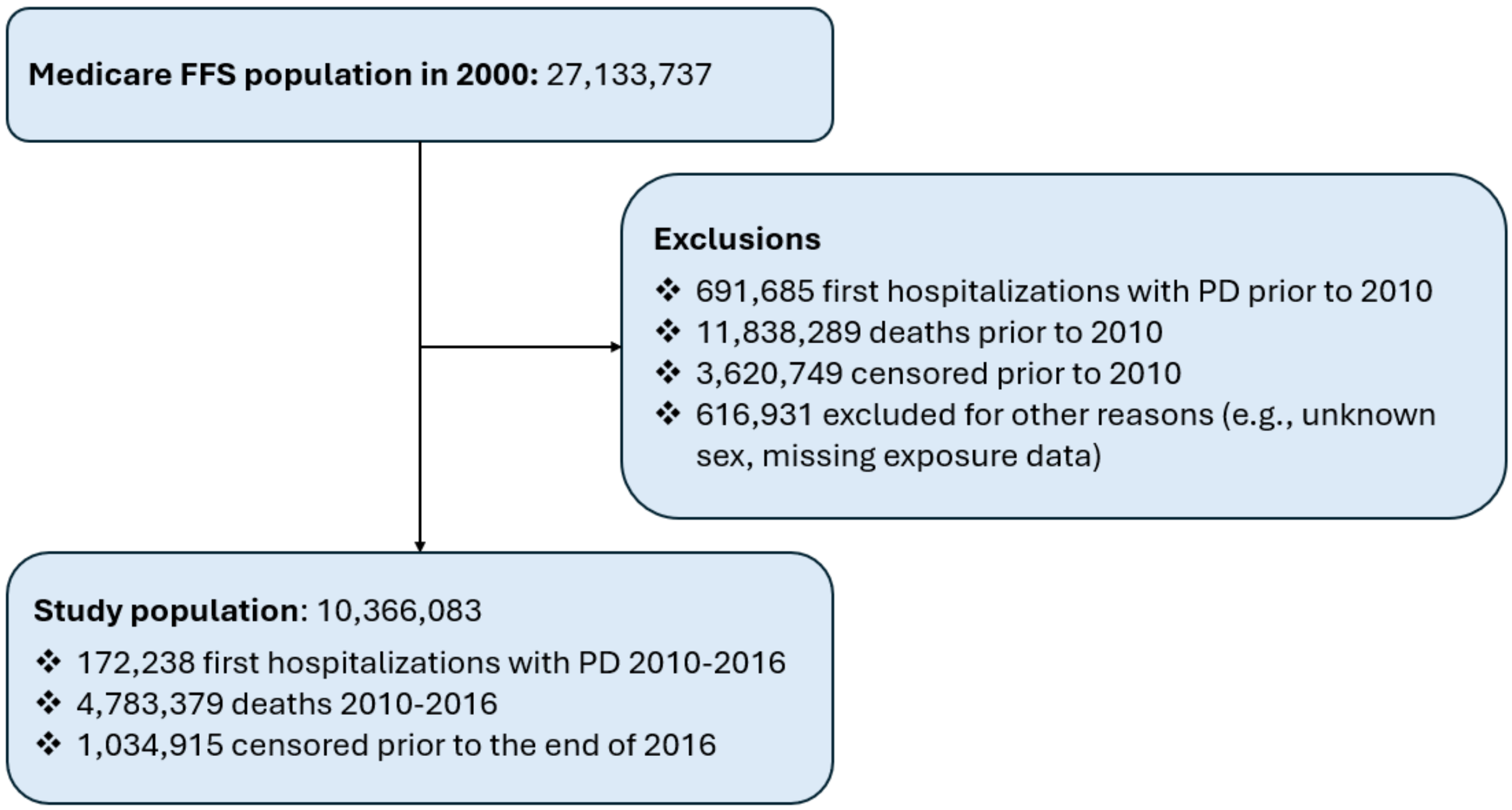

Supplement Figure 1. **Study population inclusion/exclusion diagram.** Beneficiaries were required to be alive, continuously enrolled in Medicare Fee-for-Service, and free of hospitalizations with PD during the lead-up period (2000-2009).

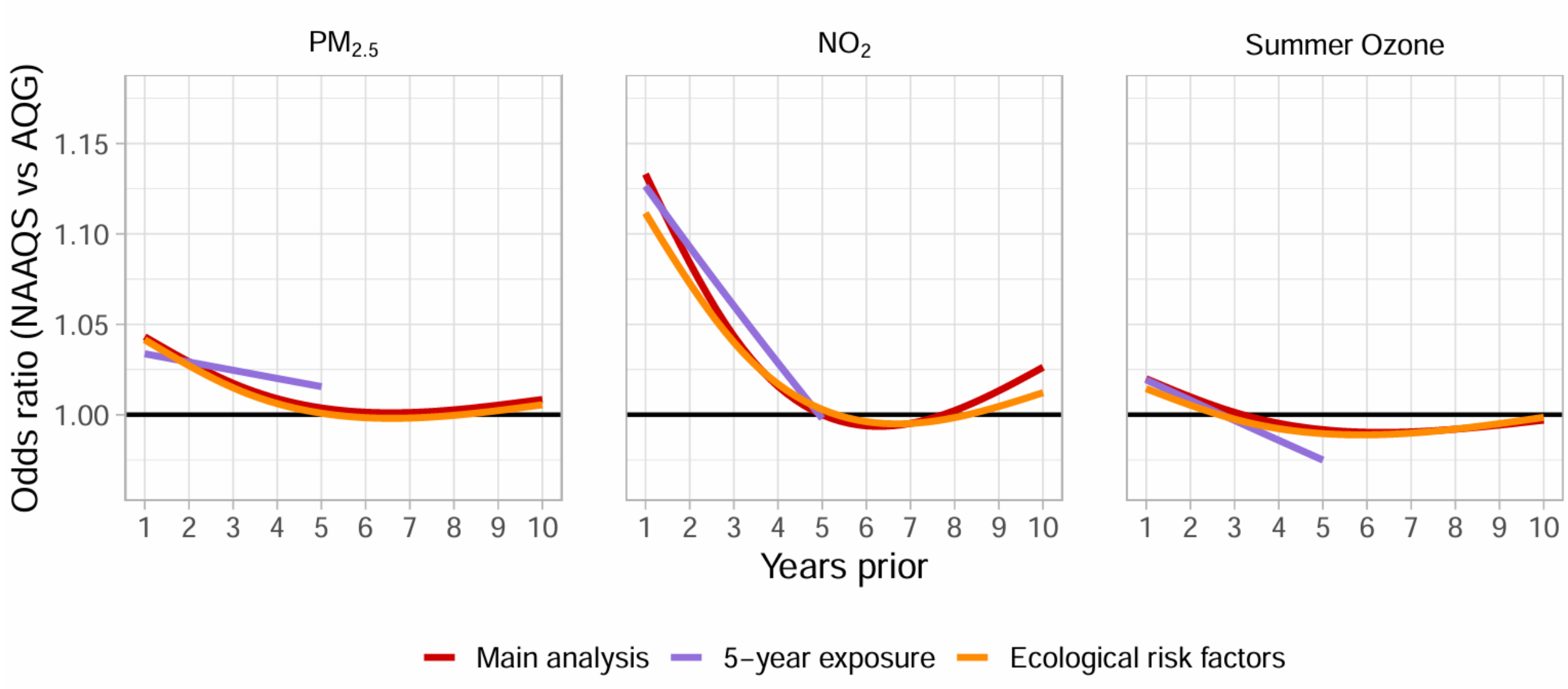

Supplement Figure 2. Distributed lag linear model odds ratios for sensitivity analyses. Sensitivity models using a 5-year exposure history only appear in purple, while models including additional ecological risk factor variables appear in orange. Main model results appear in red.

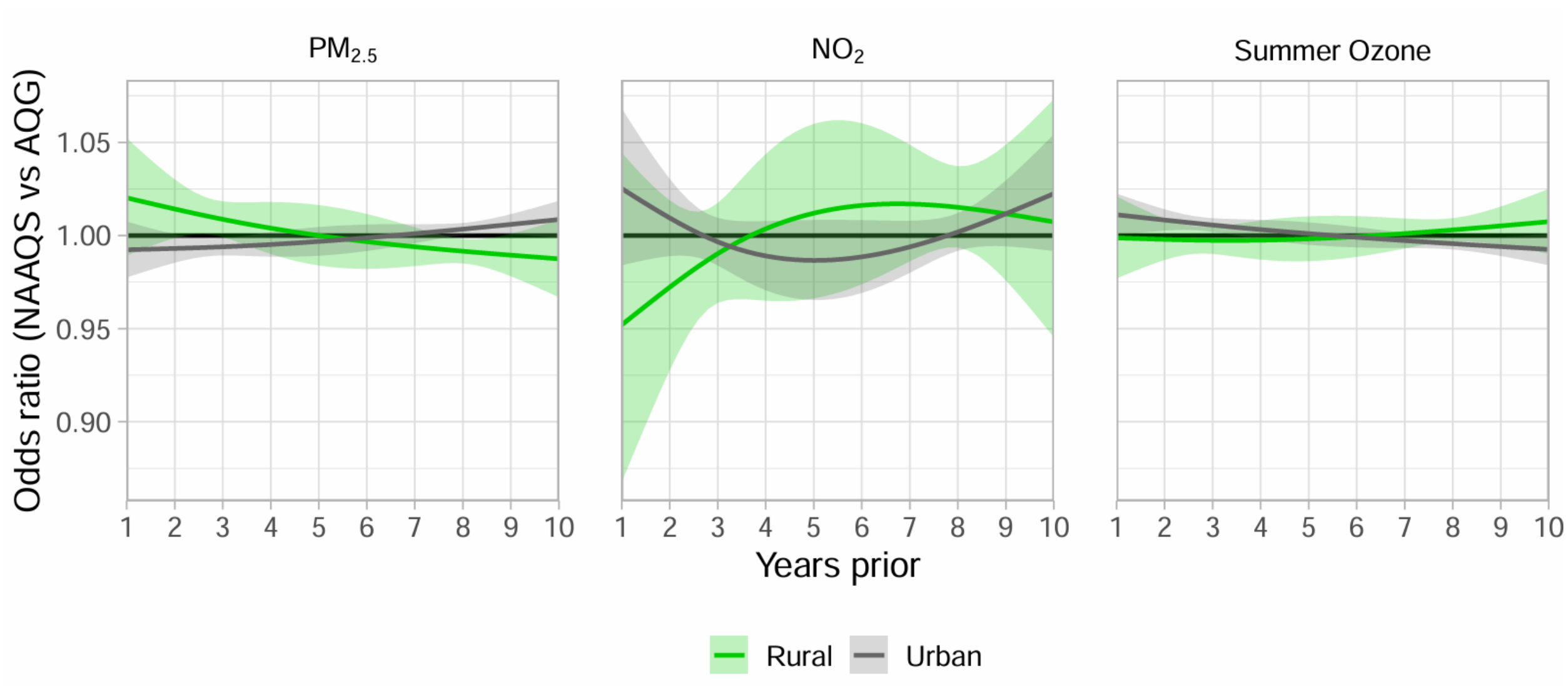

Supplement Figure 3. Lag-response linear relationship comparing annual air pollutant exposure to NAAQS versus AQG levels, stratified by urbanicity. Plotted are distributed lag odd ratios (y-axis) with 95% confidence intervals (shaded area) from one to ten prior years (x-axis) due to a change from AQG to NAAQS levels (Table 2) in each pollutant.

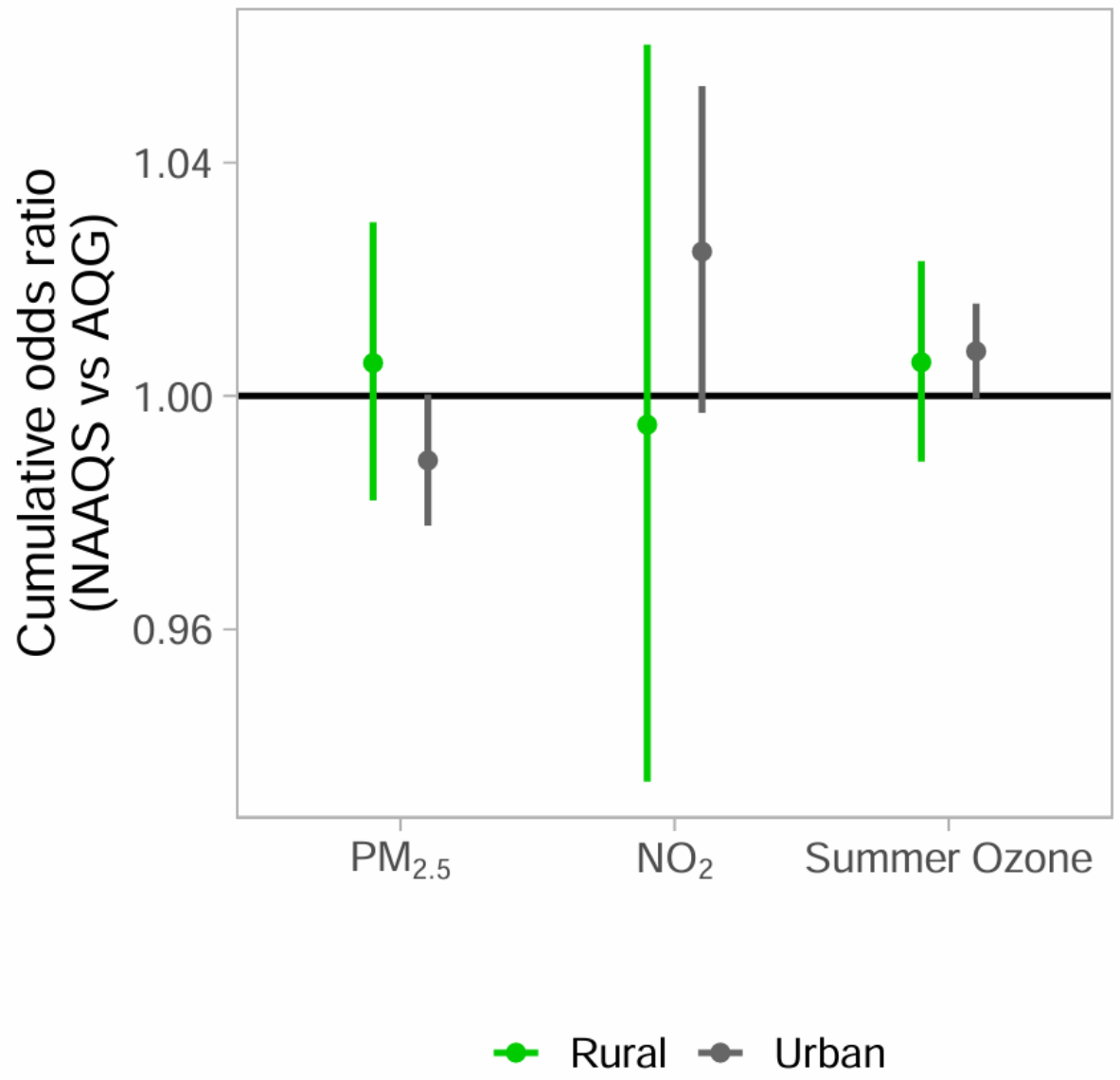

Supplement Figure 4. 10-year cumulative association between air pollutant exposure and risk of hospitalization with PD, stratified by urbanicity. Plotted are cumulative odds ratios for each pollutant with 95% confidence intervals, comparing odds at NAAQS versus AQG (Table 2) for each pollutant. Cumulative ORs are calculated by summing the distributed lag estimates over the entire exposure time span.
